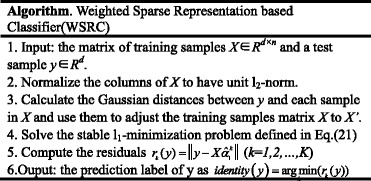# Improved protein-protein interactions prediction via weighted sparse representation model combining continuous wavelet descriptor and PseAA composition

**DOI:** 10.1186/s12918-016-0360-6

**Published:** 2016-12-23

**Authors:** Yu-An Huang, Zhu-Hong You, Xing Chen, Gui-Ying Yan

**Affiliations:** 1 Department of Computing, Hong Kong Polytechnic University, Hung Hom Hong Kong, China; 2 Xinjiang Technical Institute of Physics and Chemistry, Chinese Academy of Sciences, Urumqi, 830011 China; 3 School of Information and Electrical Engineering, China University of Mining and Technology, Xuzhou, 221116 China; 4 Academy of Mathematics and Systems Science, Chinese Academy of Sciences, Beijing, 100010 China

**Keywords:** Protein-protein interactions, Protein sequence, Continuous wavelet transform, Sparse representation based classifier

## Abstract

**Background:**

Protein-protein interactions (PPIs) are essential to most biological processes. Since bioscience has entered into the era of genome and proteome, there is a growing demand for the knowledge about PPI network. High-throughput biological technologies can be used to identify new PPIs, but they are expensive, time-consuming, and tedious. Therefore, computational methods for predicting PPIs have an important role. For the past years, an increasing number of computational methods such as protein structure-based approaches have been proposed for predicting PPIs. The major limitation in principle of these methods lies in the prior information of the protein to infer PPIs. Therefore, it is of much significance to develop computational methods which only use the information of protein amino acids sequence.

**Results:**

Here, we report a highly efficient approach for predicting PPIs. The main improvements come from the use of a novel protein sequence representation by combining continuous wavelet descriptor and Chou’s pseudo amino acid composition (PseAAC), and from adopting weighted sparse representation based classifier (WSRC). This method, cross-validated on the PPIs datasets of *Saccharomyces cerevisiae*, *Human* and *H. pylori*, achieves an excellent results with accuracies as high as 92.50%, 95.54% and 84.28% respectively, significantly better than previously proposed methods. Extensive experiments are performed to compare the proposed method with state-of-the-art Support Vector Machine (SVM) classifier.

**Conclusions:**

The outstanding results yield by our model that the proposed feature extraction method combing two kinds of descriptors have strong expression ability and are expected to provide comprehensive and effective information for machine learning-based classification models. In addition, the prediction performance in the comparison experiments shows the well cooperation between the combined feature and WSRC. Thus, the proposed method is a very efficient method to predict PPIs and may be a useful supplementary tool for future proteomics studies.

## Background

In this post-genomic era, protein, as the major component of organism, is widely studied because of its important role in nearly all cell functions including DNA transcription and replication, metabolic cycles, and signaling cascades. Researches show that many functions of complex biological systems seem to be more closely determined by their interactions rather than their individual components. Therefore, the protein-protein interaction networks have been dawning increasing research attentions and interests. Moreover, the recent advance in practical applications in drug discovery comes to be a promotion factor for studies on PPIs which provides great insights into mechanisms of human diseases. Efforts have been devoted to the development of experimental methods for detecting PPIs and constructing protein interaction networks, such as yeast two-hybrid (Y2H) [1, 2] screens, tandem affinity purification (TAP) [3], mass spectrometric protein complex identification (MS-PCI) [3] and other high-throughput biological techniques for PPIs detection. However, experimental methods are expensive, time-consuming and tedious. Meanwhile experimentally identified PPIs are usually associated with high rates of both false positive and false negative results. For the sake of detecting larger fraction of the whole PPI network and utilizing the valuable and vast biological data provided by experimental methods, there is a growing need to develop computational methods capable of identifying PPIs.

A number of computational approaches haven been proposed for detecting PPIs based on various data types, such as genomic information, protein domain and protein structure information [4]. However, these methods are limited by the need of prior information about proteins, and the accuracies of them are sensitive to the reliability of the prior information. In addition, the exponential growth of newly discovered protein sequences is accumulated in numerous different types of databases. Therefore, it is significant to develop sequence-based PPI predicting systems mining information directly from amino acid sequences. Many researchers have engaged in trials to establish sequence-based system for predicting PPIs and have gained some preliminary result. To solve this problem, Zhou et al. [5] proposed an approach combing support vector machine and local protein sequence descriptors which account for the interactions between sequentially distant amino acid residues. When applied to predicting yeast PPIs, this method yielded a promising accuracy of 88.56%. Najafabadi et al. [6] found similarity in codon usage is a strong predictor for expressing proteins and got a 75% increase in sensitivity in his experience considering codon usage. Shi et al. [7] explored a kind of descriptor named correlation coefficient transformation and used support vector machine and this method adequately considers the neighboring effect and the level of correlation coefficient.

Computational systems for predicting pairwise protein interactions usually rely on two main components: feature extraction and machine learning model. Efficient feature descriptors are capable of mining useful information and normalizing different-length proteins to the same size. Furthermore, effective feature extraction methods can lead to an improvement in prediction performance. Until now, a number of feature extraction approaches based on protein sequence have been proposed and most of them consider the sequence order effect. In fact, employing graphic approaches to mine proteins’ information would be of great novelty. In this work, we adopt a novel descriptor named CW-LBP and show it is sufficient to reveal the complicated relations between protein interactions and their amino acid sequences. This sequence representation first encodes the protein sequence as a numerical sequence by substituting each amino acid with a specific proteins’ physicochemical property. Then, Meyer continuous wavelet transformation is employed to represent a protein sequence as an image. Finally, an image texture descriptor, Local Binary Pattern Histogram Fourier (LBP-HF) is used to extract features. In order to describe a protein in a discrete model which could provide comprehensive information, Chou’s pseudo amino acid composition (PseAAC) is employed as another kind of feature descriptor. PseAAC is a popular protein descriptor using the first 20 factors to reflect components of 20 conventional amino acid (AA) compositions and λ additional factors to reflect the influence of sequence order.

As the second step of computational methods for predicting PPIs, a wide range of machine learning models have been applied in previous works. However, the popular classifiers such as SVM [8, 9] and neural network [10] need much effort to adjust the optimal parameters. Recently, Sparse Representation based Classification (SRC) comes to be a new technique in study of face recognition for its excellent ability against illumination variations, occlusions, and random noise. Matching the feature descriptors extracted by the proposed graphic-based features (i.e., LBP-HF descriptors), SRC would be an ideal classification model. As indicated in the study of [11], Weighted Sparse Representation based Classifier (WSRC), a variant of basic SRC, additionally consider the local information of each training samples and therefore have a strong classification ability surpassing original SRC. In addition, WSRC needs little manual invention to adjust the optimal parameters, which is a significant character for the vast data volume of various protein sequence sets. Thus, WSRC algorithm is used as the machine learning tool to make the final prediction based on the extracted feature sets.

In this study, we report a novel computational method for predicting protein-protein interactions based on amino acid sequences by using the classifier of WSRC and the combined features consisting of CW-LBP and PseAAC descriptors. Firstly, each protein is transformed into a CW image deriving from amino acid sequence and then CW-LBP features are extracted from these images using LBP-HF texture descriptor. Secondly, for a more comprehensive representation for protein sequences, we extracted the Chou’s pseudo amino acid composition of each sample and merged it with CW-LBP descriptor as the whole feature set. By doing this, our feature representation of one protein would own 216 dimensions of which 176 come from CW-LBP descriptor and 40 is the Chou’s PseAA composition. Finally, WSRC is utilized to deal with the classification. To evaluate the performance, the proposed approach is applied to three different PPI data sets: *Saccharomyces* c*erevisiae, Human,* and *H.pylori.*

## Results

### Evaluation measures

To evaluate the performance of the proposed method, we use five-fold cross validation and a couple of evaluation measures such as the overall prediction accuracy (Accu.), sensitivity (Sens.), precision (Prec.) and Matthews correlation coefficient (MCC) in this study. These criteria are defined as follows:

1 $$ Accuracy=\frac{TP+TN}{TP+FP+TN+FN} $$

2 $$ Sensitivity=\frac{TP}{TP+FN} $$

3 $$ PE=\frac{TP}{TP+FP} $$

4 $$ MCC=\frac{TP\times TN-FP\times FN}{\sqrt{\left(TP+FN\right)\times \left(TN+FP\right)\times \left(TP+FP\right)\times \left(TN+FN\right)}} $$

where true positive (TP) denotes the number of true samples which are predicted correctly; false negative (FN) is the number of true samples predicted to be non-interacting pairs incorrectly; false positive (FP) is the number of true non-interacting pairs predicted to be PPIs falsely, and true negative (TN) is the number of true non-interacting pairs predicted correctly. Furthermore, the receiver operating characteristic (ROC) curve was also used to evaluate the performance of proposed method. Summarizing ROC curve in a numerical way, the area under an ROC curve (AUC) was computed. A higher AUC value means a better result performed.

### Assessment of prediction ability

For the sake of impartiality, we set the same corresponding parameters (*σ* = 1.5, *ε* = 0.00005) for WSRC when we explored using the proposed method to predict PPIs of *Saccharomyces cerevisiae* and *H.plpori* dataset. In order to minimize the overfitting of the prediction model and test the robustness of the proposed method, 5-fold cross-validation was used in our experiments. In 5-fold cross-validation, dataset would be divided into five parts which four of them are used for training and the rest one of them is used for testing. By this way, five models were generated from the original dataset.

**Fig. 1:**
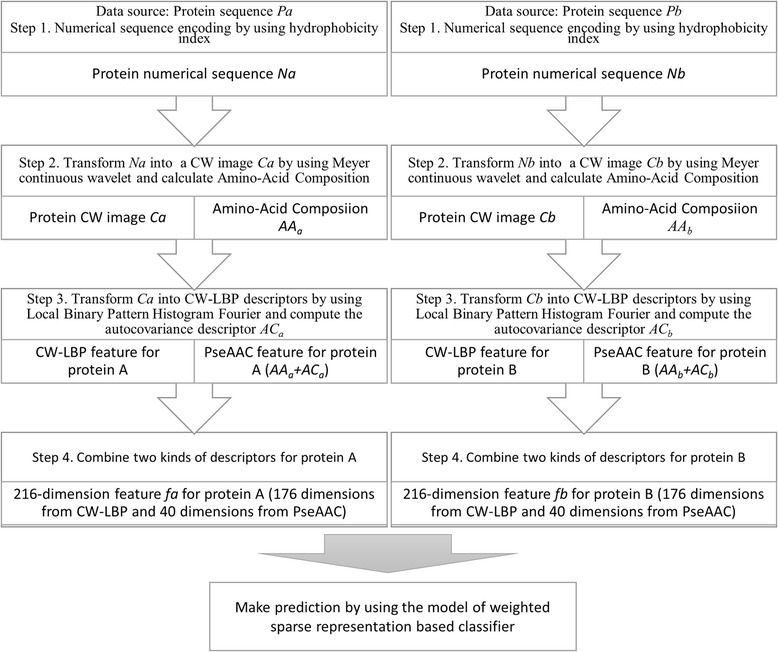
The flowchart for the feature extraction process

**Fig. 2:**
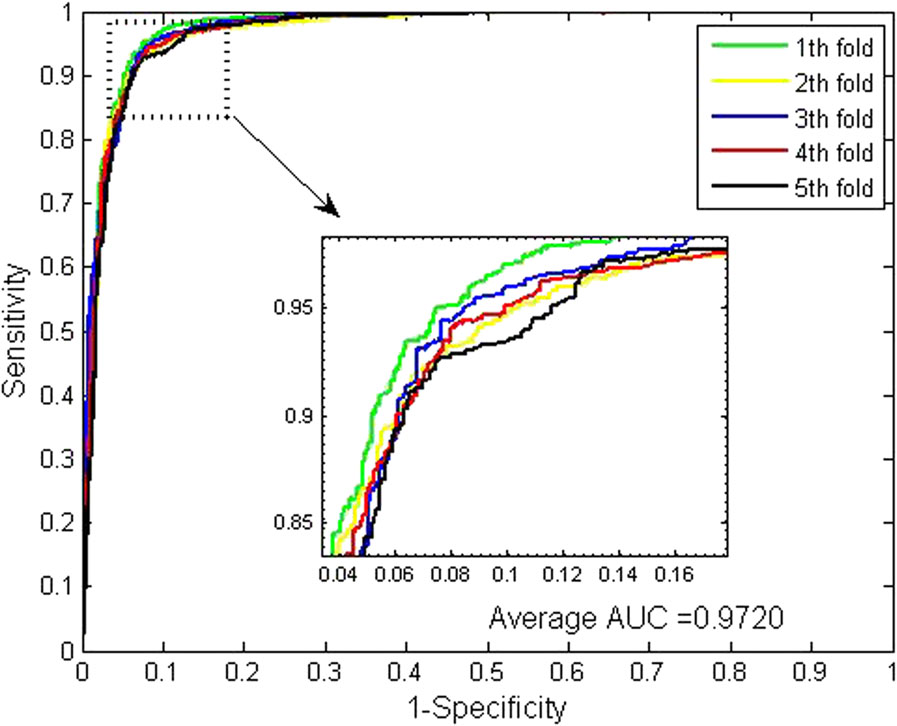
ROC curves from proposed method result for *Saccharomyces cerevisiae* PPIs dataset

The prediction results of SRC prediction models with continuous wavelet features and PseAA composition are shown in Table 1 and Table 2. For all five models of *Saccharomyces cerevisiae* dataset, the prediction accuracies are ≥ 91.83%, the precisions are ≥ 95.01%, and the sensitivities are 87.64%. For the five models of *H.pylori* dataset, the prediction accuracies are ≥ 83.30%, the precisions are ≥ 78.25% and the sensitivities are ≥ 89.27%. In order to better evaluate the practical prediction ability of the proposed model, we also calculate the MCC and AUC values (see Figs. 1 and 2). From Table 1 and Table 2, it can be observed that the averages of MCC and AUC score of *Saccharomyces cerevisiae* dataset are 86.09% and 97.20% respectively.

**Table 1:** 5-fold cross validation result obtained in predicting Yeast PPIs dataset

Test set	Accu.(%)	Prec.(%)	Sen.(%)	MCC(%)	AUC(%)
1	93.43	96.98	89.93	87.70	97.70
2	92.27	95.01	89.39	85.71	96.99
3	92.36	96.62	87.64	85.81	97.39
4	92.62	95.65	89.19	89.30	97.09
5	91.83	95.10	87.95	84.94	96.80
Average	92.50 ± 0.59	95.87 ± 0.89	88.82 ± 0.98	86.09 ± 1.02	97.20 ± 0.35

**Table 2:** 5-fold cross validation result obtained in predicting H.pylori PPIs dataset

Test set	Accu.(%)	Prec.(%)	Sen.(%)	MCC(%)	AUC(%)
1	85.03	82.18	90.67	74.28	92.36
2	83.30	78.25	91.12	71.91	91.33
3	84.34	80.00	90.46	73.44	91.84
4	84.17	82.99	89.27	72.83	92.04
5	84.59	78.85	91.11	73.79	91.96
Average	84.28 ± 0.64	80.45 ± 2.07	90.54 ± 0.77	73.25 ± 0.92	91.91 ± 0.37

**Fig. 3:**
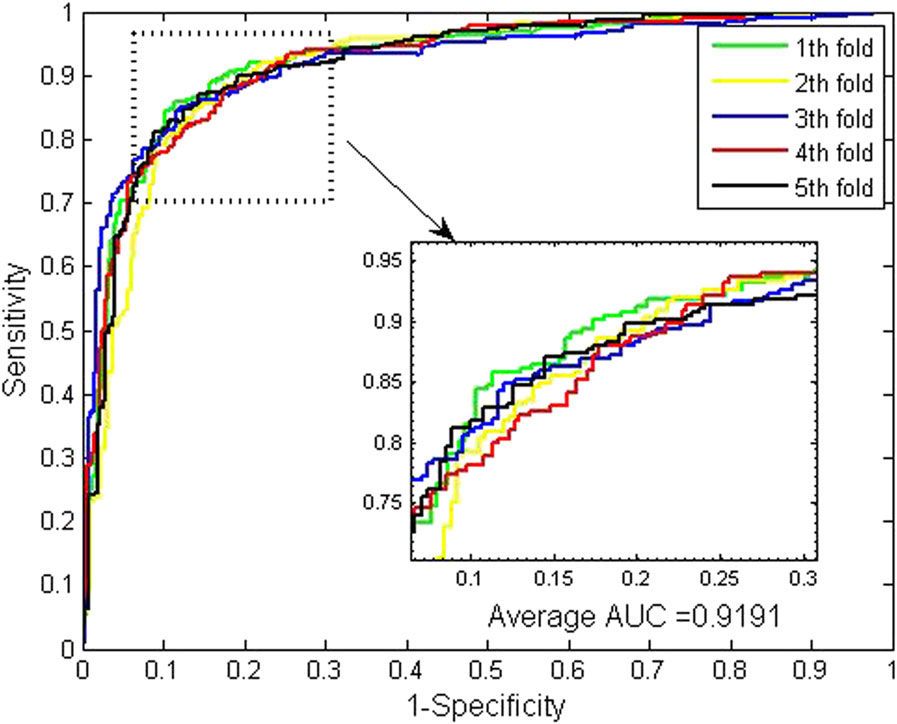
ROC curves from proposed method result for *H.pylori* PPIs dataset

When predicting the PPIs of *H.pylori* dataset, the averages of MCC and AUC come to be 73.25% and 91.91% (see Fig. 3). Further, we can see that our method achieved a stable performance with the low standard deviations of accuracy, precision, sensitivity, MCC and AUC as 0.59%, 0.89%, and 0.98%, 1.02% and 0.35% respectively.

### Comparison with SVM-based method

Many machine learning models haven been proposed for predicting the protein-protein interactions and one of the most popular classifiers is support vector machine (SVM). To further evaluate the proposed method, using the same feature extraction method, we explored SVM for predicting PPIs of *Human* dataset to compare with the performance of WSRC. Here, two parameters *c* and *g* of SVM were optimized by using a grid search method. Parameters *c* and *g* were set to be 10 and 15 respectively. We also used 5-fold cross-validation in these experiments. The results performed by WSRC and SVM are shown in Table 3.

**Table 3:** 5-fold cross validation result obtained in predicting Human PPIs dataset

Classification model	Testing set	Accu.(%)	Prec.(%)	Sen.(%)	MCC(%)
WSRC	1	95.53	99.14	91.17	91.35
	2	95.89	98.61	92.59	92.06
	3	95.22	99.19	91.09	90.86
	4	95.83	98.74	92.31	91.94
	5	95.22	99.04	91.08	90.85
	Average	95.54 ± 0.32	98.95 ± 0.25	91.65 ± 0.74	91.41 ± 0.58
SVM	1	87.68	87.60	85.64	78.26
	2	87.56	88.04	85.18	78.10
	3	87.68	88.66	86.14	78.38
	4	90.07	89.54	89.31	82.05
	5	87.63	89.92	84.05	78.23
	Average	88.13 ± 1.09	88.75 ± 0.98	86.06 ± 1.97	79.00 ± 1.71

**Fig. 4:**
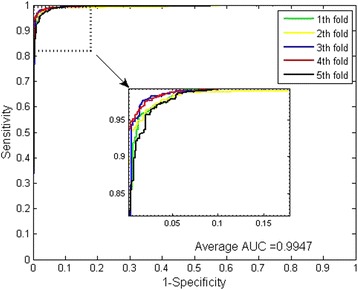
ROC curves from proposed method result for Human PPIs dataset

**Fig. 5:**
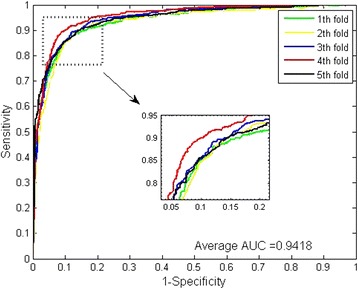
ROC curves from SVM-based method result for Human PPIs dataset

It can be observed that WSRC yielded good results with averages of accuracy, precision, sensitivity and MCC as high as 95.54%, 98.95%, 91.65% and 91.41% respectively. When using SVM for the prediction, the averages of accuracy, precision, sensitivity and MCC come to be 88.13%, 88.75%, 86.06% and 79.00% respectively, lower than the results from the WSRC-based model. From the ROC curves of Fig. 4 and Fig. 5, we can see that the average AUC score of WSRC model was 99.47%, higher than that of SVM model. In addition, it can be noticed that the standard deviations of accuracy, precision, sensitivity, MCC yielded by WSRC model are as low as 0.32%, 0.25%, 0.74% and 0.58%, lower than those of SVM model which are 1.09%, 0.98%, 1.97% and 1.71% respectively. Analyzing all these results, we consider the proposed method based on WSRC is superior to the SVM-based method.

### Comparison with other methods

Many methods have been proposed for predicting PPIs. Here, we compare the prediction ability of the SRC prediction model using continuous wavelet transform descriptors and PsaAA composition with the existing methods. From Table 4, we can see the results of 5-fold cross-validation from different methods on the *Saccharomyces cerevisiae* dataset. Most averages of accuracy, precision and sensitivity yielded by other methods are lower than the results yielded by the proposed method. Meanwhile, we can see that the proposed method is more stable than other methods with relatively low standard deviations of accuracy, precision, sensitivity and MCC as 0.59%, 0.89%, 0.98% and 1.02% respectively. The average results performed by other methods on *H.pylori* dataset are shown in Table 5.

**Table 4:** Performance comparison of different methods on the Yeast dataset

Model	Method	Accu.(%)	Prec.(%)	Sen.(%)	MCC(%)
Guos’ work [23]	ACC	89.33 ± 2.67	88.87 ± 6.16	89.93 ± 3.68	N/A
	AC	87.36 ± 1.38	87.82 ± 4.33	87.30 ± 4.68	N/A
Zhous’ work [5]	SVM + LD	88.56 ± 0.33	89.50 ± 0.60	87.37 ± 0.22	77.15 ± 0.68
Yangs’ work [24]	Cod1	75.08 ± 1.13	74.75 ± 1.23	75.81 ± 1.20	N/A
	Cod2	80.04 ± 1.06	82.17 ± 1.35	76.77 ± 0.69	N/A
	Cod3	80.41 ± 0.47	81.86 ± 0.99	78.14 ± 0.90	N/A
	Cod4	86.15 ± 1.17	90.24 ± 1.34	81.03 ± 1.74	N/A
Proposed method	WSRC	92.50 ± 0.59	95.87 ± 0.89	88.82 ± 0.98	86.09 ± 1.02

**Table 5:** Performance comparison of different methods on the H.pylori dataset

Model	Accu.(%)	Prec.(%)	Sen.(%)	MCC(%)
Phylogenetic booststrap [25]	75.80	80.20	69.80	N/A
HKNN [26]	84.00	84.00	86.00	N/A
Signature products [13]	83.40	85.70	79.90	N/A
Boosting [7]	79.52	81.69	80.37	70.64
Proposed method	84.28	80.45	90.54	73.25

## Discussion

In the proposed model, the protein features are extracted by using the transformations of numerical sequences, continuous wavelet and Local Binary Pattern Histogram Fourier. (see Fig. 1) This feature extraction method is mainly based on the assumptions that the information of protein sequences can provide enough information for predicting protein-protein interactions and the fact that the hydrophobicity character of protein influences the protein interacting process. To retain comprehensive information by feature extraction, there are two kinds of descriptors, namely CW-LBP and PseAAC, adopted to capture the continuous and discrete information, respectively. In addition, in order to combine with the CW-LBP feature well and to develop a prediction model which need little manual intervention, the classification method of weighted sparse representation-based classifier is used to make the final prediction.

It is worthwhile to highlight several aspects of the proposed approach based on the experiments results here. (1) The outstanding prediction performance shows that continuous wavelet transformation can cooperate well with the Local Binary Pattern Histogram Fourier for protein feature extraction. (2) The comparison result of WSRC versus SVM demonstrates that WSRC can be well combined with graph-based feature extraction method and the use of CW-LBP may help WSRC give a full play to its function. (3) It is worth noting that WSRC could yield stable and satisfactory prediction performance by keeping the same parameters in all experiment. Compared with other conventional classifiers including SVM, WSRC has a valuable advantage that it doesn’t need much manual intervention to adjust the optimal parameters and therefore has great potential to be applied to the large-scale prediction for new PPIs. (4) It is known that approaches using ensemble classifier usually achieve more accurate and robust performance than the methods using single classifier. However, using the single classifier, our proposed model obtains good performance similar to those obtained by the methods using ensemble classifier such as boosting. From these comparisons, it is demonstrated that the WSRC-based model combining the continuous wavelet transform descriptor and PseAA composition can improve the prediction accuracy compared with current state-of-the-art classification mothods.

## Conclusions

The growing demand for PPIs knowledge is promoting the development of studies on computational methods for predicting PPIs. In this paper, we propose a new PPIs prediction model only using the information of protein sequences. Since hydrophilic interaction plays an important role in the process of protein interactions, we consider the hydrophobic property of amino acids in the process of feature extraction by transforming protein sequences into numerical ones. We then adopted continuous wavelet descriptors and Chou’s pseudo amino acid composition, which aims at capturing the continuous and discrete information from the hydrophobic sequences. Besides, weighted sparse representation based classifier was used as the sample classification model due to its advantages of low manual intervention in parameter adjustion and good cooperation with features.

Results obtained from our experiments have shown that it is a good attempt to represent proteins using graphic texture extraction approaches. Our proposed method is feasible and effective. When performed on the *Saccharomyces cerevisiae*, *Human* and *H.pylori* datasets, the proposed method achieved promising results with high average accuracies of 92.50%, 95.54% and 84.28% respectively.

## Methods

### Gold standard datasets

We verify the proposed method on a high confidence *Saccharomyces* c*erevisiae* PPIs data set. It is gathered from publicly available database of interacting proteins (DIP). We removed those protein pairs which have ≥40% sequence identity or whose lengths are less than 50 residues. Consequently, we got the remaining 5594 protein pairs which construct the positive data set. Besides, 5594 additional protein pairs whose sub-cellular localizations are different were chosen to build the negative data set. As a result, the whole data set consists of 11188 protein pairs of which half are from the positive samples and half are from the negative samples.

To demonstrate the generality of the proposed method, we also verify our approach on two other types of PPIs data sets. The first dataset is collected from the Human Protein References Database (HPRD). We removed those protein pairs which have ≥25% sequence identity. Finally, we used the remaining 3899 protein-protein pairs of experimentally verified PPIs from 2502 different human proteins to comprise the golden standard positive dataset. For golden standard negative dataset, we then followed the previous work [12] assuming the proteins in different subcellular compartments do not interact with each other. By this way, we finally obtained 4262 protein pairs from 661 different human proteins as the negative dataset. Consequently, the *Human* dataset is constructed by 8161 protein pairs. The second PPI dataset is composed of 2916 *Helicobacter pylori* protein pairs (1458 interacting pair and 1458 non-interacting pairs) as described by Martin et al. [13].

### Continuous wavelet transformation

Wavelets are very effective and popular descriptors for all kinds of applications. *Li* et al. [14] firstly used wavelets features to descript protein sequence, which offer a novel insight into mining proteins information. Compared with Fourier transform, wavelet transform has a completely different merit function. It uses functions which are localized in both the real and Fourier space while Fourier transform decomposes the input signal into sines and cosines. As an implementation of the wavelet transform, continuous wavelet transform (CWT) use arbitrary scales and almost arbitrary wavelets. Reinforcing the traits due to the redundancy tends, continuous analysis is often easier to interpret.

Since wavelet encoding could only deal with numerical representation, we first encoded the protein sequence substituting every amino acid with protein’s hydrophobicity index which is offered by AAindex dataset. Then, based on these numerical sequences, we applied Meyer continuous wavelet to produce proteins’ CW images. In continuous wavelet transformation, a digital signal can be decomposed into many groups of coefficients by different scales. These groups of coefficients can represent characteristics in both time domain and frequency domain. In this work, we considered 100 decomposition scales using Meyer continuous transformation in the feature extraction process. CWT can be formulized as follow:

5 $$ {W}_f\left(a,b\right)=\frac{{\displaystyle \int f(t)\psi \Big(\frac{t-b}{a}}\Big)dt}{\sqrt{a}} $$

where *a* is the scale parameter and *b* is the shift factor; *ψ(t)* is wavelet core; *f(t)* is the digital signal sequence; and *W*_*f*_ *(a,b)* is the result of inner product operation between *f(t)* and *ψ(t)*.

### Local binary pattern histogram fourier (LBP-HF)

Local binary pattern (LBP) is a particular case of the Texture Spectrum model and a popular type of feature used for classification in computer vision. This texture descriptor computes specific values of each pixel based on the information of its neighborhood. In addition, researches have pointed out that when combined with the Histogram of oriented gradients (HOG) descriptor, LBP would obtain an improvement of detection performance on some datasets. LBP can be formulized as follow:

6 $$ LBP\left({x}_c,{y}_c\right)={\displaystyle \sum_{m=0}^{p-1}s\left({i}_n-{i}_c\right)\times {2}^n} $$

7 $$ s\left({i}_n-{i}_c\right)=\left\{\begin{array}{l}1\begin{array}{ccc}\hfill \hfill & \hfill if\hfill & \hfill {i}_n-{i}_c\ge 0\hfill \end{array}\\ {}0\begin{array}{ccc}\hfill \hfill & \hfill if\hfill & \hfill {i}_n-{i}_c<0\hfill \end{array}\end{array}\right. $$

where *i*_*c*_ denotes to the value of the centered pixel while i_n_ represents the neighbors’ values, *P* is the number of neighboring pixels. *Ahonen* et al. [15] first proposed Local Binary Pattern Histogram Fourier (LBP-HF). This method first computes a LBP histogram and then uses the discrete Fourier transform to construct rotationally invariant features from the histogram. Since this method only computes *P-1* Fast Fourier Transforms of *P* points from the LBP histogram, it has a lower overhead than LBP histogram. Here (*P = 16; R = 2*) and (*P = 8; R = 1*).

### Pseudo amino acid composition (PseAAC)

Due to the simplicity and effectiveness, the amino acid composition model comes to be a popular feature description for detecting protein attributes. For the sake of avoiding losing the sequenced-order information, Pseudo Amino Acid Composition [16] has been proposed to add additional values which can reflect the influence of sequence order. So PseAAC formed as this concatenation has stronger representation ability beyond the traditional AAC. Several studies [17] have shown that many useful descriptors could be produced when Amino-Acid Sequence is coupled with other information related to the physiochemical properties of amino acids. For this reason, we applied hydrophobicity index of amino acids to the producing of PseAAC descriptors. In this work, we adopted Autocovariance (AC) approach method which is one of the sequence-based variants of Chou’s pseudo amino acid composition.

Given a protein sequence *P = (p*_*1*_ *, p*_*2*_ *… p*_*N*_ *)* and fixing a physicochemical property *d*, the *20* values of PseAAC descriptor are composed of Amino-Acid Composition (AA) which can be symbolized as follow:

8 $$ AA(i)=\frac{n(i)}{N},\begin{array}{cc}\hfill \hfill & \hfill i\in \left[1,\dots, 20\right]\hfill \end{array} $$

where *n(i)* counts the number of occurrences of a given amino acid in a protein sequence of length N.

The next *20* values of PseAAC descriptor are autocovariance descriptor which is *AC* ^*d*^ ∈ *ℜ* ^20 + *m*^ and symobolized as follow:

9 $$ A{C}^d(i)={\displaystyle \sum_{k=1}^{N-i+20}\frac{\left( value\left({p}_k,d\right)-{\mu}_d\left)\cdot \right( value\left({p}_{k+i-20},d\right)-{\mu}_d\right)}{\sigma_d\cdot \left(N-i+20\right)}}\begin{array}{cc}\hfill \hfill & \hfill i\in \left[21,\dots, 20+m\right]\hfill \end{array} $$

where *value(i,d)* is a function returning the value of the property *d* for the amino acid *i*; *μ*_*d*_ and *σ*_*d*_ denote the normalized mean and the variance of d on the *20* amino acids:

10 $$ {\mu}_d=\frac{1}{20}{\displaystyle \sum_{i=1}^{20} value\left(i,d\right)} $$

11 $$ {\sigma}_d=\frac{1}{20}{\displaystyle \sum_{i=1}^{20}{\left( value\left(i,d\right)-{\mu}_d\right)}^2} $$

### Weighted sparse representation based classification (WSRC)

Recently, sparse representation based classification (SRC) algorithm has been developed and successfully used for classification, becoming a hot topic of pattern recognition and computer vision. Supposing that there is a training sample matrix X∈*R* ^*d×n*^ which represents n training samples and d-dimensional feature vectors, SRC assumes that there are sufficient training samples belonging to the *kth* class and makes up ${X}_k=\left[{l}_{k1}\cdots {l}_{k{n}_k}\right]$ where *l*_*i*_ and *n*_*k*_ denote the label of *ith* sample and the sample number of *kth* class respectively. Thus, sample matrix *X* could be rewritten as *X* = [*X*_*1*_…*X*_*K*_]. Given any test sample y∈*R* ^*d*^, it can be approximately represented as the linear combination of kth-class training samples:

12 $$ y={\alpha}_{k,1}{l}_{k,1}+{\alpha}_{k,2}{l}_{k,2}+\cdots +{\alpha}_{k,{n}_k}{l}_{k,{n}_k} $$

When represented as the linear combination of all the training samples, *y* could be symbolized as follow:

13 $$ y=X{\alpha}_0 $$

where ${\alpha}_0={\left[0,\cdots, 0,{\alpha}_{k,1},{\alpha}_{k,2}\cdots {\alpha}_{k,{n}_k},0,\cdots, 0\right]}^T$. Here, since the nonzero entries in *α*_*0*_ are only associated with the *kth* class, *α*_*0*_ would be sparse if the class number of samples is large.

For SRC, many efforts are devoted to search a vector *α* such that Eq. (9) is satisfied and the l _0_-norm of *α* is minimized. This can be described as:

14 $$ {\widehat{\alpha}}_0= \arg \min {\left\Vert \alpha \right\Vert}_0\kern0.5em  subject\ to\kern0.5em y=X\alpha $$

The formulation (10) is a NP-hard problem which can be achieved but difficult to solve precisely [18]. However, the theory of compressive sensing [19] reveals that if *α* is sparse enough, we can solve the related convex l_1_-minimization problem instead of solving the solution of l_0_-minimization problem directly:

15 $$ {\widehat{\alpha}}_1= \arg \min {\left\Vert \alpha \right\Vert}_1\kern0.5em  subject\ to\kern0.5em y=X\alpha $$

Dealing with occlusion, we can extend the Eq. (11) to the stable l_1_-minimization problem:

16 $$ {\widehat{\alpha}}_1= \arg \min {\left\Vert \alpha \right\Vert}_1\kern0.5em  subject\ to\kern0.5em \left\Vert y-X\alpha \right\Vert \le \varepsilon $$

where *ε* > 0 denotes to the tolerance of reconstruction error. Eq. (12) can be solved via standard linear programming methods [20].

After obtaining the sparsest solution ${\widehat{\alpha}}_1$, we can assign test sample y to class *k* by the following rule:

17 $$ \underset{k}{ \min }{r}_k(y)=\left\Vert y-X{\widehat{\alpha}}_1^k\right\Vert, \kern0.5em k=1\dots K $$

where $X{\widehat{\alpha}}_1^k$ is the reconstruction which is constructed by training samples of class *k* and *K* is the class number of the whole samples. Given all this, traditional SRC represents a test sample as a sparse combination of training sample and assigns it to the class which minimizes the residual between itself and $X{\widehat{\alpha}}_1^k$.

However, researches [21, 22] have shown that in some case, locality structure of data is more essential than sparsity. In addition, the traditional SRC fails to guarantee to be local. To overcome this problem, weighted sparse representation based classifier (WSRC) expands SRC by combining the locality structure of data with sparse representation. It is well-known that an appropriate kernel function which maps the samples into a high dimensional feature space by a nonlinear mapping can change the samples’ distribution and make the samples from one class more similar. For this reason, WSRC evaluates the similarity of two samples by employing Gaussian-kernel based distance which can be symbolized as follow:

18 $$ {d}_G\left(x,y\right)={e}^{-{\left\Vert x-y\right\Vert}^2/2{\sigma}^2} $$

where *x,y*∈*R* ^*d*^ denote two samples and *σ* is the Gaussian kernel width. By doing this, WSRC penalizes the distance between a test sample and each training data and preserves the similarity while seeking the sparse linear representation. Given a test sample y and a training sample matrix *X*, WSRC solves the following weighted l_1_-minimization problem:

19 $$ {\widehat{\alpha}}_1= \arg \min {\left\Vert W\alpha \right\Vert}_1\kern0.5em  subject\ to\kern0.5em y=X\alpha $$

and specifically,

20 $$ diag(W)={\left[{d}_G\left(y,{x}_1^1\right),\dots, {d}_G\left(y,{x}_{n_k}^k\right)\right]}^T $$

where *W* is a block-diagonal matrix of locality adaptor, which uses the Gaussian distances as the weights of training samples; *n*_*k*_ denotes the sample number of training set in class *k*. Dealing with occlusion, we solve the stable l_1_-minimization problem of Eq. (19) as follow:

21 $$ {\widehat{\alpha}}_1= \arg \min {\left\Vert W\alpha \right\Vert}_1\kern0.5em  subject\ to\kern0.5em \left\Vert y-X\alpha \right\Vert \le \varepsilon $$

where *ε > 0* is the tolerance value.

The WSRC algorithm is summarized as follows: